# An assessment of high-resolution wind speeds downscaled with the Weather Research and Forecasting Model for coastal areas in Ghana

**DOI:** 10.1016/j.heliyon.2021.e07768

**Published:** 2021-08-13

**Authors:** Denis E.K. Dzebre, J. Ampofo, Muyiwa S. Adaramola

**Affiliations:** a Department of Mechanical Engineering, Kwame Nkrumah University of Science and Technology, Kumasi, Ghana; b The Brew-Hammond Energy Centre, Kwame Nkrumah University of Science and Technology, Kumasi, Ghana; c Faculty of Environmental Sciences and Natural Resources Management, Norwegian University of Life Sciences, Ås, Norway

**Keywords:** Wind mapping, Wind resources assessment, WRF, Dynamic downscaling, Ghana, West African coast

## Abstract

Ghana produces over 50% of its electrical energy demands from fossil-fuelled thermal plants. To increase the proportion of renewable energy in the national energy generation, a Renewable Energy Master Plan (REMP) which seeks, among others, to shift the country's national energy generation capacity towards more renewable energy sources has been developed. The REMP noted that inadequate data on renewable energy sources such as wind is one of the challenges to achieving this target. In this regard, this paper assessed the open-source Weather Research and Forecasting Model, as a tool for generating wind resource data. The WRF model is often used to downscale meteorological datasets for wind resources assessments. However, due to diverse model options, performance assessments are required to establish the accuracy and suitability of a model configuration for an application in an area. This paper assessed the performance of a Weather Research and Forecasting Model configuration that is based on previous verification studies. In evaluation, data accuracy benchmarks were generally met by the downscaled wind data. A wind map that was generated was observed to be generally accurate and better than the previous 2001 wind map for Ghana. It is presumed that the configuration is suitable for wind mapping activities for the coastal areas in Ghana, and probably neighbouring countries. However, for downscaling time-series data, further studies are recommended.

## 1 Introduction

Over-dependence on electricity from thermal and hydro sources has been one of the major causes of electricity supply challenges in Ghana over the years. Thermal plants, which run on fossil fuels generated more than 50% of Ghana's electricity in 2019 [1]. Diversifying the electricity generation mix of Ghana through the development of other energy sources, including renewable sources such as wind and solar energy, has often been recommended as one of the ways of addressing the challenge [2].

Assessing the wind resource in an area is an important step towards understanding its characteristics and economic exploitation. Ground measurements of wind characteristics serve as the most reliable source of data for such assessments. However, data generated from downscaled meteorological data sets, have been used in some stages of wind resource and feasibility assessments, owing to the expensive and time-consuming nature of ground measurements. In addition, downscaled data is increasingly used in other aspects of the resource assessment process, such as the designing of ground measurement campaigns and the conducting of pre-feasibility economic assessments. Thus, Numerical Weather Prediction (NWP) models have increasingly become popular as downscaling tools for meteorological datasets. However, model assessments (or validations) are crucial in assessing the ability of NWP models to produce reliable data. In addition, model validations are also important for optimizing the model configuration for desired applications in different areas, as the models come with diverse (physics and dynamics) options whose impacts on model performance varies with climatic conditions and terrain features.

Several efforts have been made in the evaluation of Ghana's wind resources. These include some limited ground measurements of wind data in selected areas, mostly along the coast of the country [3] and the development of a national wind map in 2001 with the proprietary Mesoscale Atmospheric Simulation System (MASS) model. However, in addition to the limited nature of these ground measurements, limited (if any) model validations were done in preparing the 2001 wind map, owing to an unavailability of adequate and reliable ground at the time [4]. Though tools like the Global Wind Atlas (GWA) [5] have become available for resource assessments, the accuracy of such tools depends on how well the models have been configured for specific applications in specific areas; something that often requires validation tests on the models. However, we found no validations of the GWA for Ghana, or its model configuration in literature. The 2019 Ghana Renewable Energy Master Plan [6] emphasizes the need for more and improved assessments on resources such as wind, if Ghana's renewable energy development goals are to be achieved.

The Weather Research and Forecasting (WRF) model [7] can play an important role in this regard, given its open-source nature and wide use in assessing wind resources the world over [8, 9, 10, 11]. With the availability of more reliable ground measurements since 2001, a series of model sensitivity studies for an area in Ghana [12, 13, 14, 15] have been conducted on different parts of the WRF modelling system, from which some model options have been recommended. However, in addition to testing the different parts of the WRF model individually, the sensitivity studies had other limitations; they covered up to four (4) months instead of the usual one year that is commonplace in such studies, they were performed at optimum model resolutions which do not necessarily give the best model performance, and they used ground measurements from only one location for validation of results.

Against this background, this paper assessed a WRF configuration that is based on the recommendations of the sensitivity studies. In addition to being conducted at relatively high resolution, this study covered two years, while the validation studies lasted up to four months. Furthermore, the generated data was verified with ground measurements from eight locations, instead of the one location that was often used in the sensitivity studies to assess the generalizability of the configuration for other areas in the coastal regions of Ghana. Verification focused on only the coastal areas of Ghana due to data limitations. A wind map was generated from the data and compared with the existing 2001 Solar, Wind Energy Resource Assessment (SWERA) [4, 16] and the more recent GWA maps for Ghana.

## 2 Data and method

This study used version 3.8.1 of the Advanced Research WRF [7], which is the same version used in the sensitivity studies. Key features of the model and detailed descriptions of the model physics, equations and dynamics are available in [12] and [7]. The model configuration, summarized in Table 1, follows the recommendations of the prior sensitivity studies for a location on the coast of Ghana [12, 13, 14, 15]. A nesting ratio of 5 was used for the nested domains, with the final horizontal domain being 1 km. Each domain had a vertical resolution of 45 vertical pressure levels. The model top was 50 hPa with the lowest half level at approximately 28 m asl.

**Table 1:** Model configuration.

Model Version	Advanced Research WRF v3.8.1		
Initial and boundary conditions	NCEP Final Analysis (GFS-FNL): 1^0^ × 1^0^ and 6 h resolution [22].		
Land Use data	30-arc-second MODIS with lakes		
Topographical data	30-arc-second USGS GMTED2010		
Map Projection	Mercator		
Vertical Resolution	45 vertical pressure levels (automatically set), 50hPa model top		
Horizontal resolution (km^2^)	25 × 25	5 × 5	1 × 1
Domain size (grid points)	121 × 120	141 × 186	181 × 136
Temporal Resolution (Minutes)	-	20 min	10 min
Model timestep (seconds)	150		
FDDA	Analysis Nudging in Domains 1 and 2 (Disabled in the PBL)		
Parameterization Schemes			
Cloud Microphysics (MP)	Eta microphysics [23]		
Long-wave Radiation (LW-Rad)	Rapid Radiative Transfer Model [24]		
Short-wave Radiation (SW-Rad)	Dudhia [25]		
Surface Layer (SL)	Eta Similarity (Eta) [26, 27, 28]		
Land Surface Model (LSM)	Unified Noah [29]		
Planetary Boundary Layer (PBL)	Mellor-Yamada Nakanishi Niino Level 3 (MYNN3) [30]		
Cumulus	Kain-Fritsch [31] (turned off for domains 2 and 3 [7,32])		

Grid (analysis) nudging in the WRF's Four-Dimensional Data Assimilation (FDDA) system is a technique that bridges the gap between the model simulations and time-interpolated values from input data. It has been used in several studies [17, 18, 19] on wind downscaling for resource assessment purposes, as well as the sensitivity studies for an area on the coast of Ghana [12, 14]. The technique was used in this study, being applied to the two outermost domains. Nudging options and simulation length times followed recommendations from one of the sensitivity studies for an area on the coast of Ghana [14]. Nudging was disabled for levels that are automatically determined to be within the Planetary Boundary Layer (PBL) by the FDDA system, as explained by [20], following recommendations from one of the sensitivity studies [14]. The parameterization configuration, which is a combination of required parameterization schemes, and other model physics and dynamics options followed the recommendations of the most recent sensitivity study [12] for an area on the coast of Ghana. To ensure model stability, a time step was set for the outer domain according to the recommended maximum 6DX (where DX is the horizontal resolution in km of the coarsest domain) [7]. The parent time step ratio which determines the time steps for the nested domains was set according to the parent grid ratio as recommended by [21].

**Figure 1:**
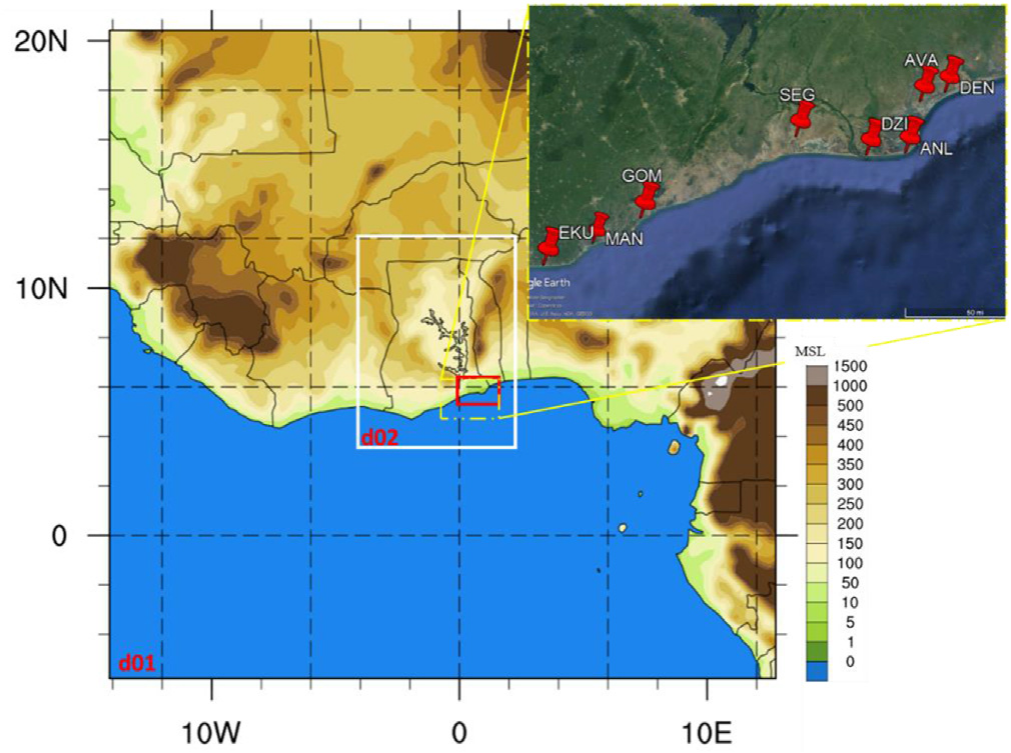
Simulation Domains and mast locations for verification data.

The simulation domains and the locations of the mast measurements used for validation are shown in Figure 1. The mast data comes from wind measurement activities carried out by the Ghana Energy Commission. The data used was measured at a height of 60-m. For two of the eight locations, the data comprised wind speeds of 10-minutes temporal resolution, while for the other locations, the data includes monthly average wind speeds. Other details are presented in Table 2.

**Table 2:** Verification data.

Location	Data temporal resolution	Period
SEG (5.872⁰N, 0.345⁰E)	10 min	Dec. 2011–Dec. 2013
ANL (5.787⁰N, 0.919⁰E)	10 min	Dec. 2012–Dec. 2013
EKU (5.208⁰N, 0.950⁰W	Monthly Average	Dec. 2011–Nov. 2012
GOM (5.446⁰N, 0.458⁰W)	Monthly Average	Dec. 2011–Nov. 2012
AVA (6.060⁰N, 1.005⁰E)	Monthly Average	Dec. 2011–Nov. 2012
DZI (5.774⁰N, 0.714⁰E)	Monthly Average	Dec. 2011–Dec. 2013
MAN (5.317⁰N, 0.700⁰W)	Monthly Average	Nov. 2012–Dec. 2013
DEN (6.112⁰N, 1.141⁰E)	Monthly Average	Nov. 2012–Dec. 2013

Post processing of the WRF simulation results followed the procedure used by [12]. However, winds were spline interpolated from the 10-m, the first half level (at approximately 28-m) winds and one higher half level (at approximately 69-m) winds to the 60-m for evaluation. In this study, the same evaluation metrics used in the previous sensitivity study on Ghana [12, 13, 14] and other similar studies [32, 33, 34, 35, 36] were used. The metrics, namely Mean Error (ME) as well its standard deviation (STDE), Standard Deviation (SD) of the observed and WRF wind speeds, Root Mean Square Error (RMSE), as well as Correlation Coefficient (CC), were compared to error benchmarks (RMSE <2 m/s, ME < ± 0.5 m/s, CC ≥ 0.7) which were considered as indicators of good model performance in similar performance evaluation studies [37, 38, 39].

## 3 Results

**Figure 2:**
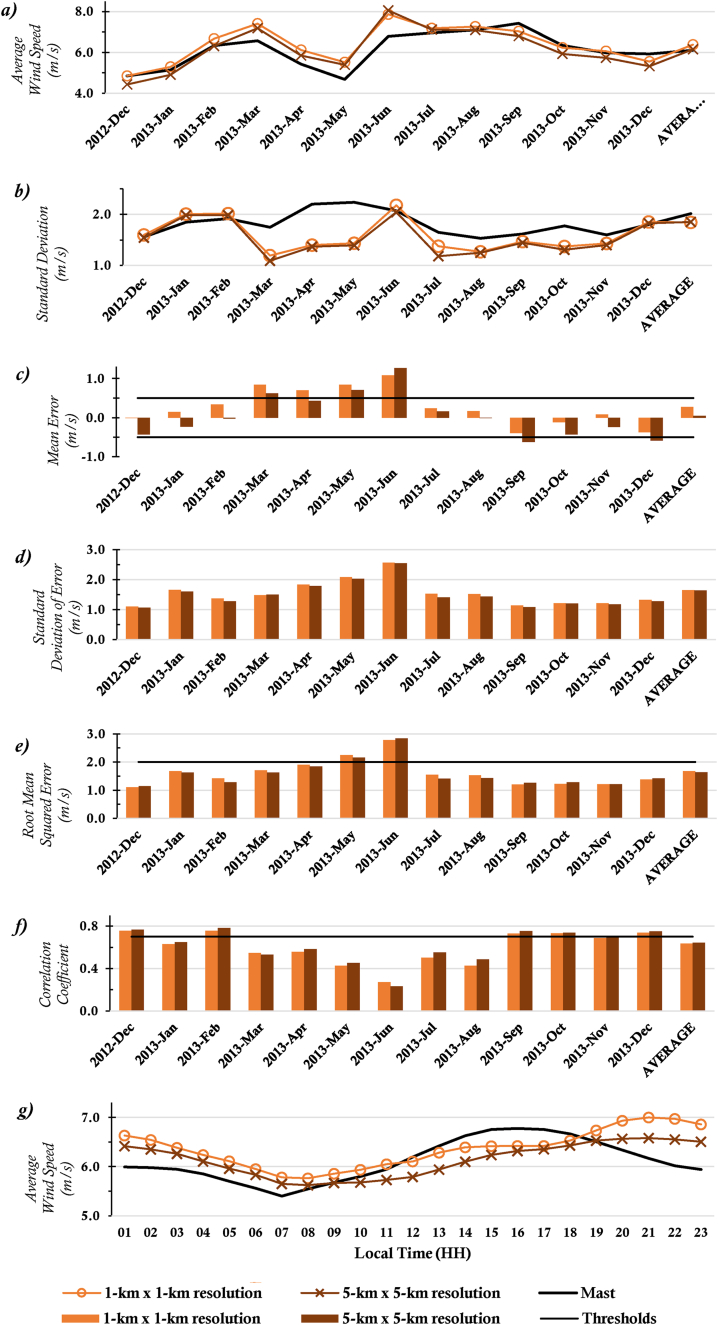
(a) Monthly Average Wind Speeds, (b) Monthly SD, (c) Monthly ME (Bias), (d) Monthly STDE, (e) Monthly RMSE, (f) Monthly CC, and (g) Diurnal Average Wind Speeds for location ANL.

**Figure 3:**
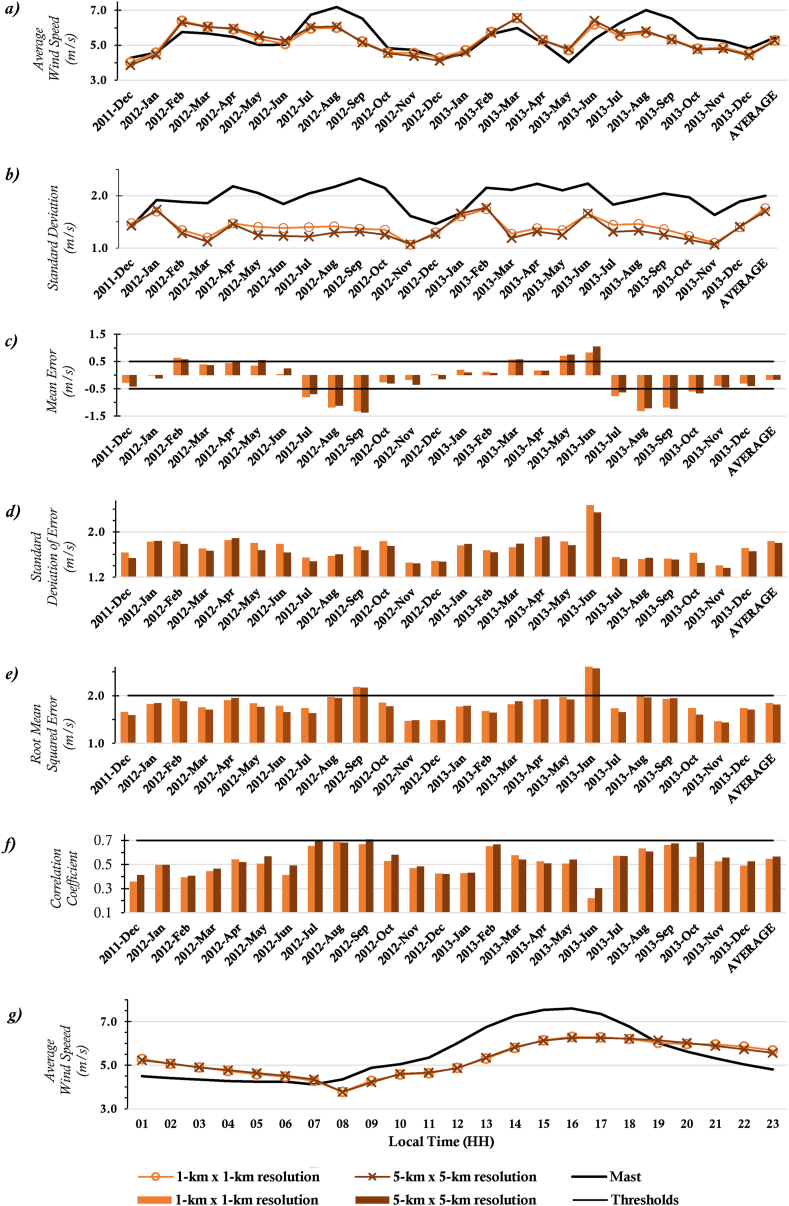
(a) Monthly Average Wind Speeds, (b) Monthly SD, (c) Monthly ME (Bias), (d) Monthly STDE, (e) Monthly RMSE, (f) Monthly CC, and (g) Diurnal Average Wind Speeds for location SEG.

**Figure 4:**
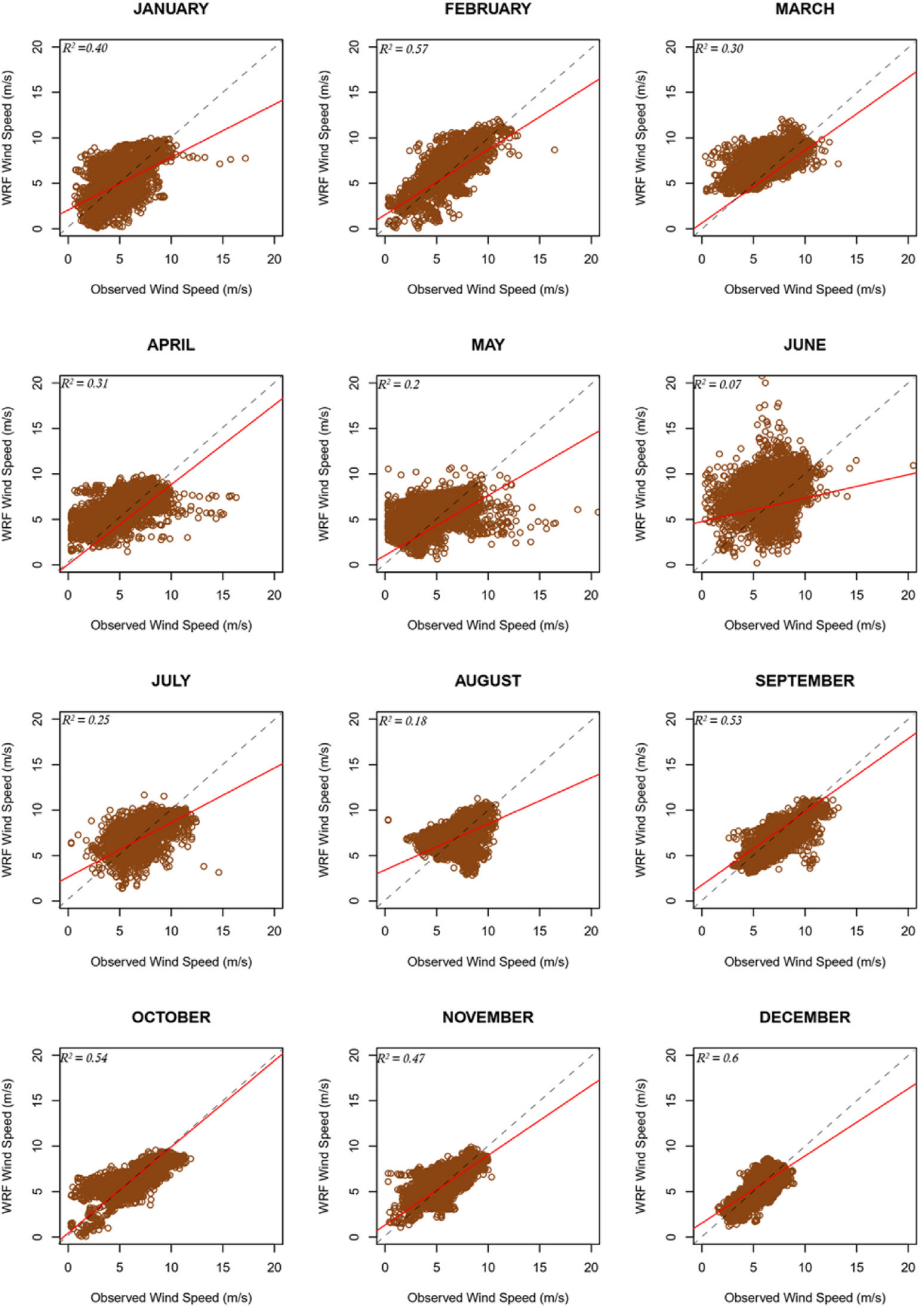
Scatter plots for average 10-minute resolution for site ANL.

**Figure 5:**
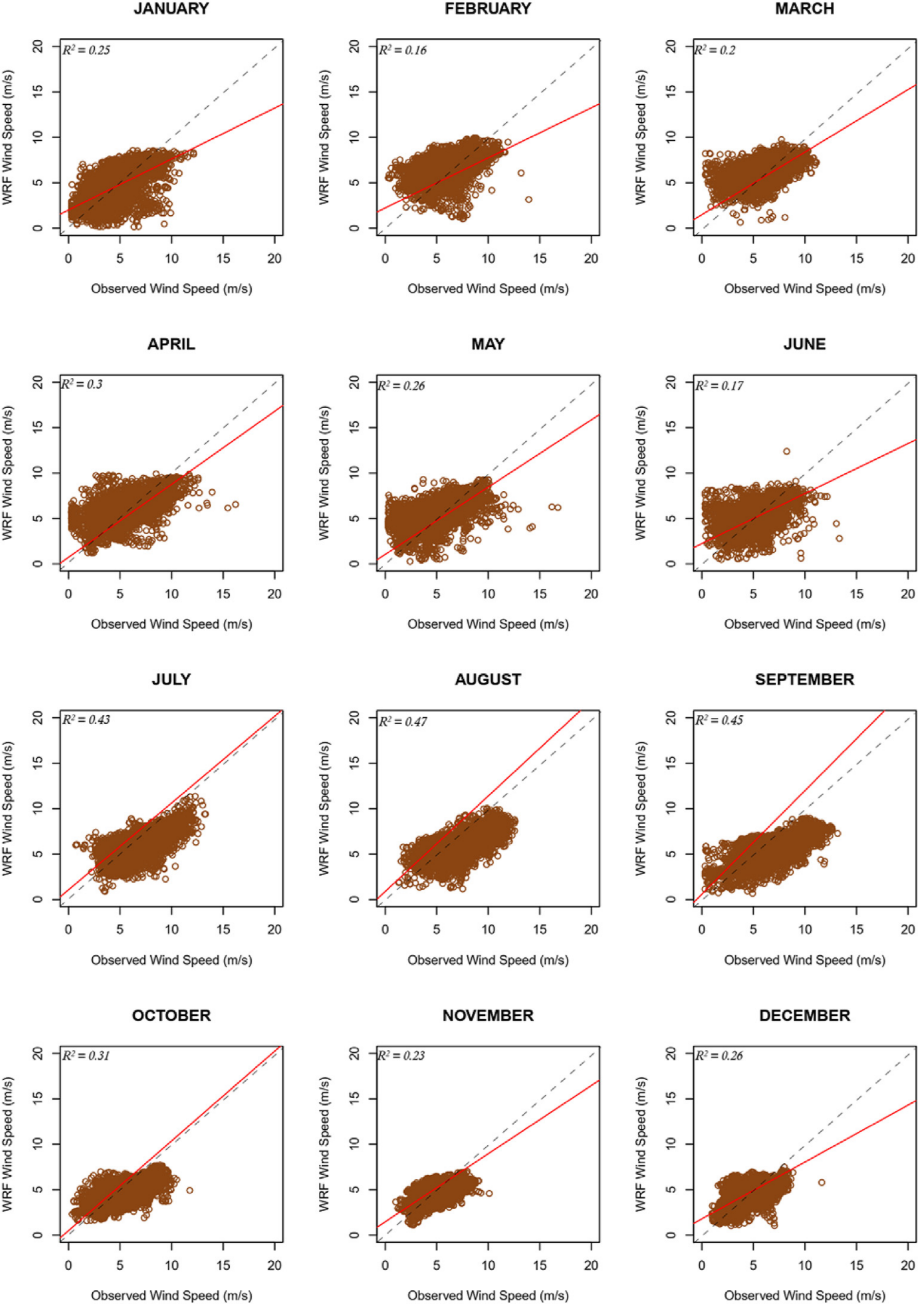
Scatter plots for average 10-minute resolution for site SEG.

Figure 2 and Figure 3 show the observed and downscaled monthly average wind speeds, monthly error metrics (i.e., ME, RMSE, SD, STDE, and CC), and the diurnal profile of wind speeds for the two locations whose observed data was of 10-minutes temporal resolution; ANL and SEG respectively. The scatter plots of these data are also presented in Figure 4 and Figure 5 for the two sites.

It can be seen from the error metric plots for location ANL that, the wind profile from the mast was largely rightly mimicked by the WRF simulations. Although generally, average wind speeds from the 1-km x 1-km resolution data were higher than averages from the 5-km x 5-km resolution data, the differences were marginal (the highest difference was less than 0.4 m/s). Observations of marginal wind peaks within 1-km x 1-km simulations are attributable to the representativeness of local variations, as high resolutions capture more details. Since the average wind speed at 1 km × 1 km resolution is usually relatively high, in months where the model underestimates wind speeds, the 1 km × 1 km resolution data usually produced relatively better absolute MEs, and vice versa. However, more often than not, the monthly MEs for data at either resolution was within the acceptable threshold (<±0.5 m/s), likewise the overall average ME. With the other error metrics, there were again marginal differences between the metrics for the data at either resolution. The monthly RMSEs met the threshold (<2.0 m/s) in all the months, except May and June 2013. The CC threshold (>0.7) was not mostly met. The diurnal profile of the wind speeds is also consistent with that from a verification study [12] for this location. Generally, the observed wind speeds are more dispersed as compared to the WRF wind speeds, especially for months in the rainy season, as can be seen from the SD plots. However, overall dispersion for observed and WRF speeds are quite similar. Furthermore, consistent with trends in the results, dispersion of the WRF wind speeds, and wind speed Mean errors irrespective of data resolution are similar (see SD and STDE plots).

Similar results were obtained for location SEG. Again, the resolution had marginal impacts on the average wind speeds and error metrics of the downscaled data. SEG, as an inland location has its low-level wind flow impeded by surface friction, as such the wind speeds are quite uniform in both resolutions used and have relatively less speeds than the coastal stations. This results in relatively less variation in the MEs when compared to those at the ANL location. However, like the case of location ANL, average metrics of data at both resolutions were often within acceptable thresholds, except for CC. The RMSE for June 2013 at this location too exceeded the acceptable threshold. Night-time winds for this location too were overestimated and vice versa during the day (Figures 2f and 3f). There is a likelihood that these observations emanate from the choice of planetary boundary layer and radiation parameterization schemes that were employed.

Given the often-similar average metrics obtained with data at either resolution, validation for the remaining locations (with average monthly wind speed data) were carried out with data at 5-km x 5-km resolution. And again, for most of the locations, overall average metrics were within acceptable thresholds. Results for these analyses are presented in Table 3.

**Table 3:** Monthly Error Metrics for downscaled data at 5-km x 5-km for 6 other locations.

	2011 Dec	2012 Jan	2012 Feb	2012 Mar	2012 Apr	2012 May	2012Jun	2012Jul	2012 Aug	2012Sep	2012 Oct	2012 Nov	2012 Dec	2013 Jan	2013 Feb	2013 Mar	2013 Apr	2013 May	2013Jun	2013Jul	2013 Aug	2013Sep	2013 Oct	2013 Nov	2013 Dec	Average	SD	RMSE	CC
DZI																													
Mast Mean	4.55	4.84	6.34	6.35	6.12	5.48	5.92	7.33	6.51	7.03	5.63	5.21	4.81	5.00	6.16	7.00	5.84	4.51	6.58	6.57	6.79	7.27	6.27	6	5.38	5.98	0.82	0.56	086
WRF Mean	3.92	4.64	6.91	6.42	6.55	6.37	6.48	7.64	7.37	6.42	5.43	4.81	4.33	4.87	6.15	7.05	5.71	5.23	8.11	7.03	7.01	6.64	5.74	5.55	5.17	6.06	0.82		
Bias	-0.63	-0.20	0.57	0.07	0.43	0.89	0.56	0.31	0.86	-0.61	-0.20	-0.40	-0.48	-0.13	-0.01	0.05	-0.13	0.72	1.53	0.46	0.22	-0.63	-0.53	-0.45	-0.21	0.08			
DEN																													
Mast Mean	-	-	-	-	-	-	-	-	-	-	-	4.25	4.45	4.51	5.49	5.75	4.85	4.00	5.35	6.00	6.70	6.20	5.25	4.85	4.70	5.20	0.71	0.63	0.74
WRF Mean	-	-	-	-	-	-	-	-	-	-	-	4.12	3.81	4.17	5.32	5.91	4.86	4.44	5.79	5.30	5.45	5.01	4.39	4.55	4.12	4.80	0.71		
Bias	-	-	-	-	-	-	-	-	-	-	-	-0.13	-0.64	-0.34	-0.17	0.16	0.01	0.44	0.44	-0.7	-1.25	-1.19	-0.86	-0.30	-0.58	-0.40			
MAN																													
Mast Mean	-	-	-	-	-	-	-	-	-	-	-	4.39	4.48	4.51	5.28	5.57	5.15	4.50	5.27	5.45	5.80	5.40	5.55	4.80	4.60	5.05	0.47	0.48	0.83
WRF Mean	-	-	-	-	-	-	-	-	-	-	-	3.97	3.82	4.41	5.11	5.82	4.86	4.26	5.34	5.11	5.37	4.86	4.42	4.22	4.14	4.69	0.47		
Bias	-	-	-	-	-	-	-	-	-	-	-	-0.42	-0.66	-0.10	-0.17	0.25	-0.29	-0.24	0.07	-0.34	-0.43	-0.54	-1.13	-0.58	-0.46	-0.36			
EKU																													
Mast Mean	3.92	4.12	4.78	5.22	4.57	4.47	4.19	4.90	4.76	5.14	4.89	5.06	-	-	-	-	-	-	-	-	-	-	-	-	-	4.67	0.40	0.81	0.42
WRF Mean	3.45	3.85	5.69	5.46	5.24	5.09	5.16	6.02	6.01	5.03	4.40	3.70	-	-	-	-	-	-	-	-	-	-	-	-	-	4.93	0.40		
Bias	-0.47	-0.27	0.91	0.24	0.67	0.62	0.97	1.12	1.25	-0.11	-0.49	-1.36	-	-	-	-	-	-	-	-	-	-	-	-	-	0.26			
AVA																													
Mast Mean	4.00	4.17	5.22	5.08	5.11	4.38	4.51	6.34	6.67	5.94	4.33	4.37	-	-	-	-	-	-	-	-	-	-	-	-	-	5.10	0.83	0.64	0.70
WRF Mean	3.65	4.32	5.96	5.79	5.81	5.32	5.15	5.79	5.89	4.93	4.23	4.23	-	-	-	-	-	-	-	-	-	-	-	-	-	5.09	0.83		
Bias	-0.35	0.15	0.74	0.71	0.70	0.94	0.64	-0.55	-0.78	-1.01	-0.10	-0.14	-	-	-	-	-	-	-	-	-	-	-	-	-	-0.01			
GOM																													
Mast Mean	3.82	4.00	4.61	4.77	4.65	4.25	4.08	5	5.19	4.97	4.33	4.73	-	-	-	-	-	-	-	-	-	-	-	-	-	4.54	0.40	0.59	0.66
WRF Mean	3.59	4.09	5.62	5.48	5.32	5.01	4.84	5.51	5.56	4.83	4.42	3.95	-	-	-	-	-	-	-	-	-	-	-	-	-	4.85	0.40		
Bias	-0.23	0.09	1.01	0.71	0.67	0.76	0.76	0.51	0.37	-0.14	0.09	-0.78	-	-	-	-	-	-	-	-	-	-	-	-	-	0.31			

**Figure 6:**
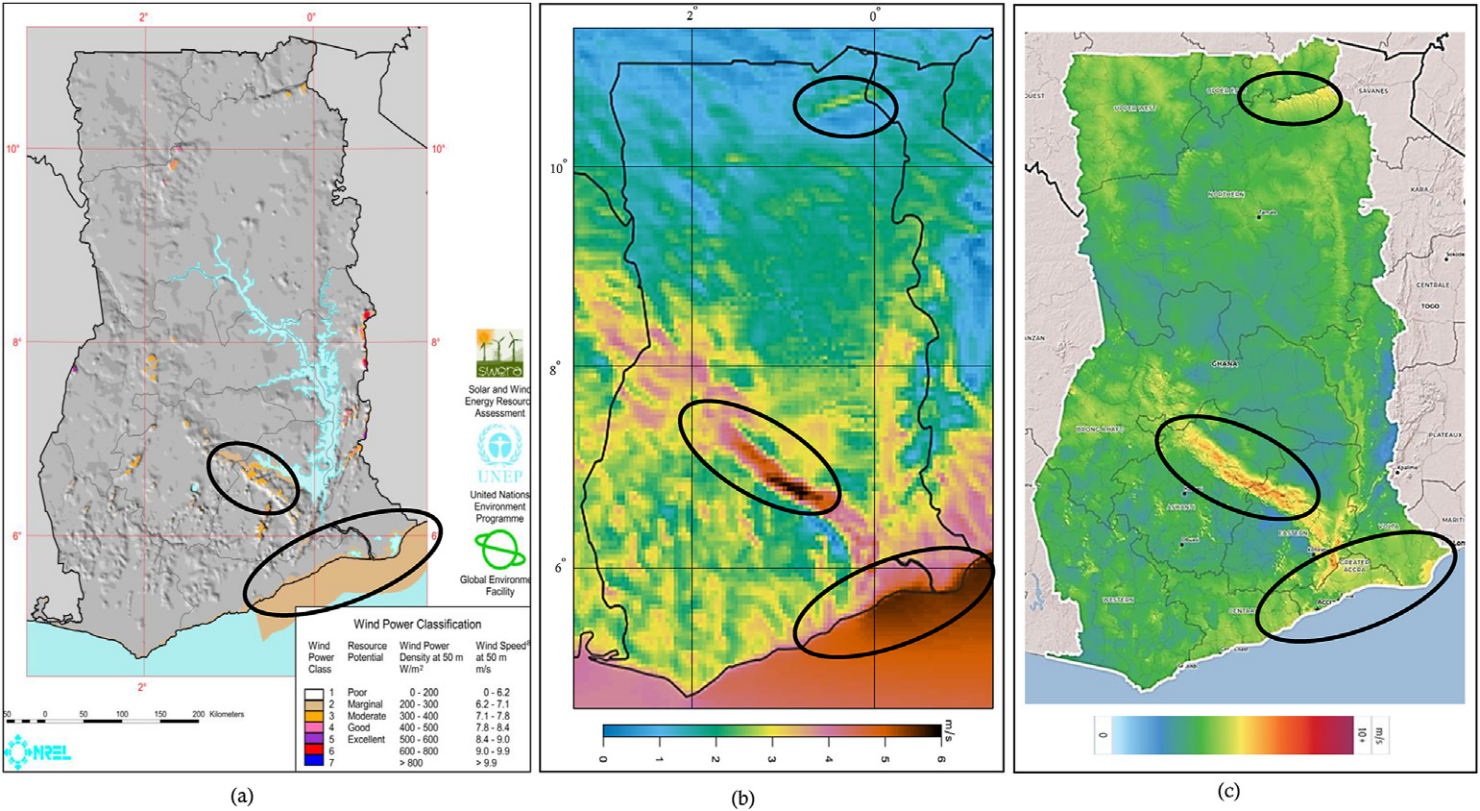
The (a) SWERA, (b) WRF, and (c) GWA wind maps at 50-m for Ghana.

**Figure 7:**
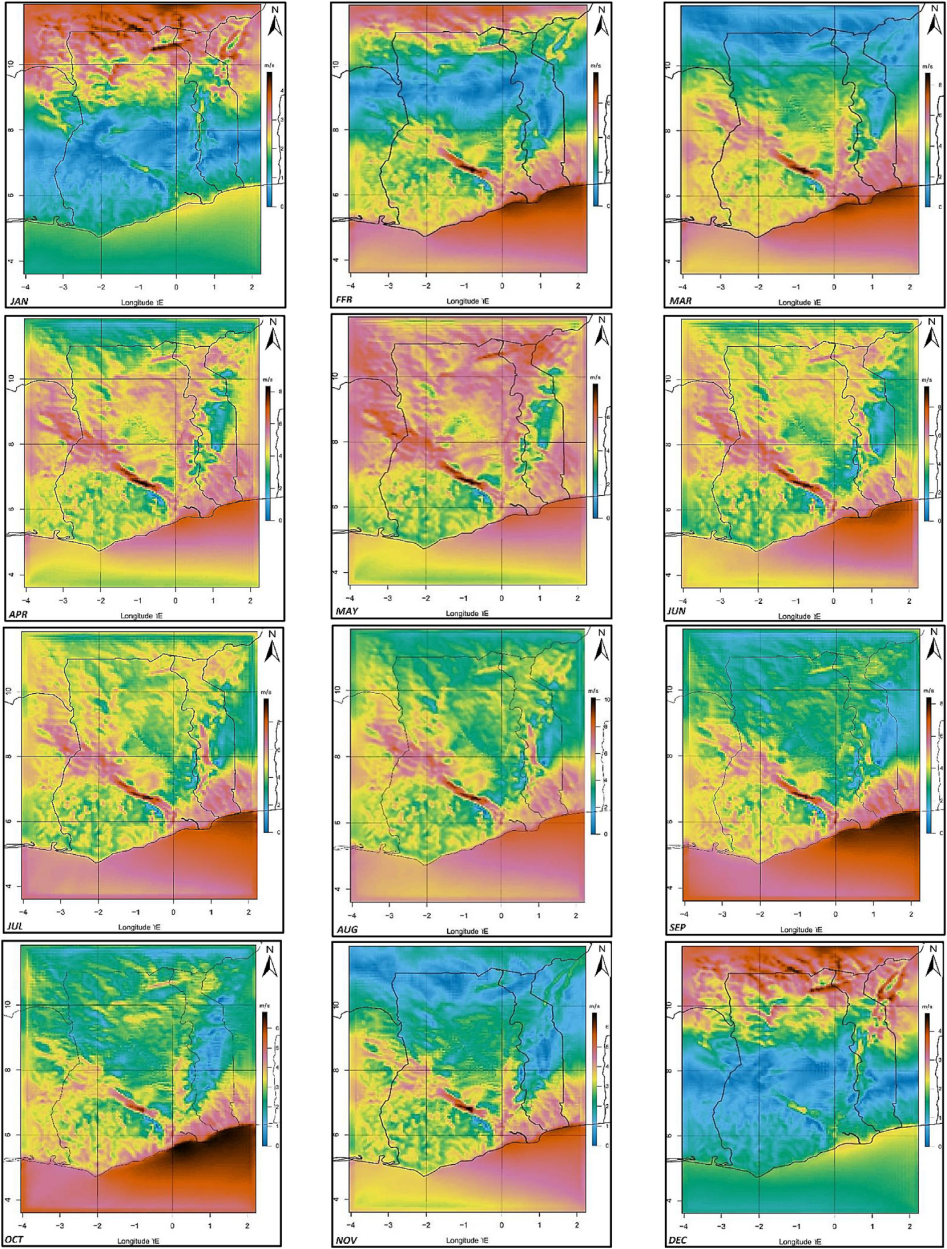
Mean monthly wind speeds at 60-m.

A wind map that was generated with averaged downscaled data spanning two years (December 2011 to December 2013) at 5-km x 5-km resolution is shown in Figure 6(b) alongside the 2001 SWERA map in Figure 6(a) and the GWA map for Ghana (Figure 6(c)). Spatial variations in monthly average wind speeds are also shown in Figure 7. It can be seen from the maps in Figure 6 that, in addition to correctly showing the same areas of generally high winds (along with the eastern coastal and the mountainous areas) as the existing SWERA map from 2001, the WRF map captures some characteristics of the average wind speeds along the coast quite better. The SWERA map estimated the minimum average wind speeds along with the eastern coastal areas as to be around 6.2 m/s at 50-m. However, ground measurements from the Energy Commission of Ghana at even higher 60-m found wind speeds at most locations in the area to be significantly lower. For instance, the minimum mean wind speed recorded at the GOM location is approximately 4.5 m/s. The average wind speeds generally increased moving eastwards. This trend of generally increasing average wind speeds eastwards is apparent on the generated WRF map. And though it was generated with data for a height of 50-m, the map represents the average wind speeds measured at 60-m quite well. This is not surprising, given that marginal differences in wind speeds at 50-m and 60-m have often been observed at certain locations along the coast of Ghana [12, 13, 15]. Spatial variation of the monthly average wind speeds with the seasons in Ghana can also be seen in Figure 7. From December there are relatively higher wind speeds in the northern regions, probably due to the harmattan winds that blow from the north until the onset of the rainy season in February/March when the Monsoon winds produce relatively higher wind speeds in the southern and middle regions.

**Figure 8:**
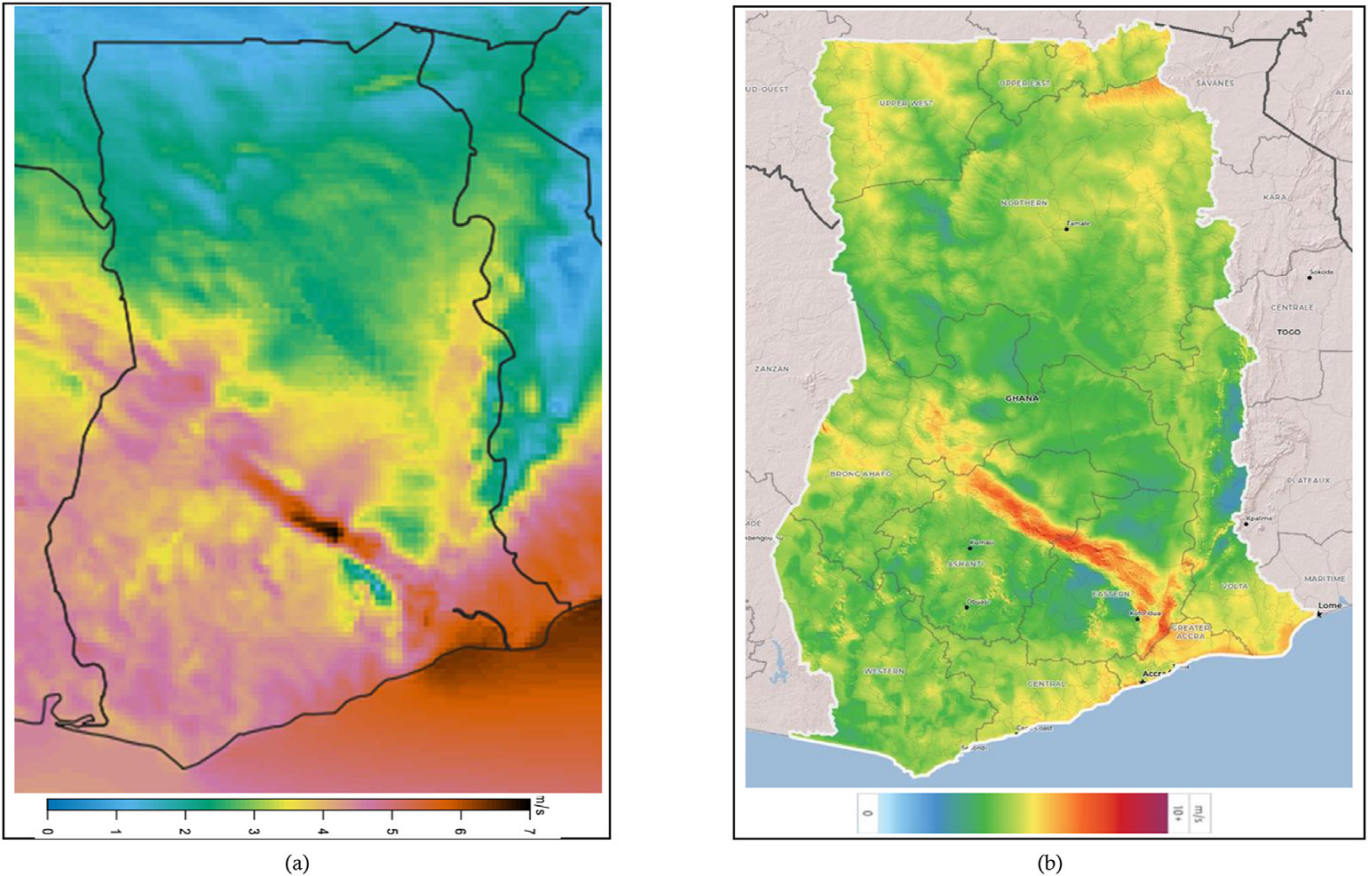
The (a) WRF and (b) GWA wind maps at 100-m for Ghana.

Compared with Ghana's 250-m GWA map in Figure 6(c), the WRF map, compares quite well, despite having average wind speeds that are marginally lower as compared to the GWA map, especially in the middle and inland areas. At 100-m, the WRF map again compares quite well with the GWA map with similarly marginally lower wind speeds especially for inland areas, as can be seen in Figure 8.

## 4 Discussion

The WRF model comes with diverse options with several possible configurations. The lack of a superior configuration over other possible candidate configurations for any application has been reported in the literature and in the prior sensitivity studies in Ghana [12, 13, 40, 41]. In fact, in the sensitivity studies, options were not recommended because they were always superior to others, but because they generally exhibited acceptable performance with varying evaluation criteria. However, the recommended options from these sensitivity studies, were tested for a limited period that were carefully selected to represent average wind speed conditions. This is one of the reasons why this study was conducted; to assess the accuracy of annual wind speeds that are downscaled with a configuration that is based on the recommended options from those tests. And per most of the monthly average error metrics, the data for different periods in the year met performance benchmarks most of the time. The results also suggest that the previously untested approach used in the sensitivity studies, (not simulating the entire year for testing, but carefully choosing a shorter test period to achieve annual average wind conditions) can recommend options that can produce acceptable average downscaled data for the entire year.

The marginal impact of data resolution on data accuracy in the coastal areas is consistent with what was realized in the sensitivity tests [12, 13]. The relatively more marginal impact of spatial resolution on average speeds for site SEG is probably due to the post-processing of the simulation data. The post-processed wind speeds (that were evaluated), were bilinearly interpolated from the simulated wind speeds at the four closest points on the simulation grid. The 5-km resolution data for site ANL was interpolated from points, some of which were further inland (as compared to the 1-km data points). Such points (from the 5-km data) probably had relatively lower wind speeds due to relatively more pronounced friction effects inland, hence the relatively lower bilinearly interpolated wind speeds for the 5-km data. In contrast, as site SEG, is a more inland site, the points from which the analyzed data were interpolated (for both resolutions) were all on land, with similar friction effects, hence the more marginal difference in metrics.

The diurnal profiles from this study are also consistent with those from the most recent sensitivity study [12] and profiles from some similar studies such as [36, 39, 42], in similarly tropical areas. As discussed by [12], the overestimation of wind before and after sunset may be due to the inability of the PBL parameterization scheme to decouple the air near the surface and the air in the night air, which is due to differences in the vertical mixing strength and entrainment of air above the PBL as explained by [36, 42].

The RMSE errors in May and June are relatively large; this is the transition period from the Pre-Monsoon to the Monsoon seasons in Ghana. Currently, there isn't enough evidence from the literature to indicate whether these observations are directly linked to any circulation features or variability in the monsoon onset. However, in this transition period, where changes in wind flow are expected, there is a chance that these circulation features account for the large errors. In a study of the Monsoon jump [43], an abrupt shift in meridional wind convergence maximum from the coast into the continental interior was observed. This shift regulates vertical moisture flux and precipitation along the coast and could again account for the variations in error observed from the study, as the model may not have fully simulated this. In addition, the error may be related to the WRF parameterization used in the simulation.

Differences between the maps can primarily be attributed to the difference in the quality of the data that was generated during the simulations. Some of these reasons include the fact that different atmospheric models were used to generate the map data, the models were run at different resolutions, and the models used different driving datasets. These and several other reasons have been found to impact the quality of atmospheric models. The accurate representation of atmospheric processes in NWP models plays an important role in the performance of NWP models, as these processes are significant in determining certain fundamental properties of the weather and climate. In NWP, this part is carried out through the physics of the model, the purpose of which is to analyse and parameterize (approximate) these processes in the model [44]. However, owing to diverse the nature of such physics options, the performance of NWPs can vary, even for the same model, when run with different options. Hence the need for sensitivity studies, to help determine the best model setup for the desired application. The SWERA map was from data generated with the Mesoscale Atmospheric Simulation System (MASS). Verification studies on the MASS could not be conducted due to a lack of adequate data at the time [4], and this could be one of the reasons for the inaccuracies that have been observed. Though the GWA was also prepared with the WRF model, we do not know its physics and dynamics configuration setup. In addition, the driving data for the GWA is different. The GWA downscaled the more recent ERA5 reanalysis dataset while this study downscaled the NCEP GFS FNL reanalysis dataset. The difference in datasets can account for some of the differences in the maps. The impact of different datasets on wind field simulations in Ghana is the focus of an ongoing study. In addition, the GWA paired the WRF model with the WASP micro scale model, which enabled it to produce data at much finer resolutions, while this study did not use a microscale model.

The relatively low inland wind speed on the WRF map can be explained by the fact that the parameterization configuration for this study is based on studies that were conducted for only the coastal areas of the country. So, the accuracy of the generated data might not exhibit the same level of accuracy in inland areas as the coastal areas. The climate in Ghana largely varies as one moves inland [45], likewise the terrain, which gets more complex as one moves inland [12]. However, it can be seen that despite that it has not been verified for inland areas, the WRF data was still able to identify inland areas with relatively better wind potential. Furthermore, the WRF wind speed averages were calculated for heights above mean sea level, and so might be higher when the elevation of the inland areas is considered in extrapolating the wind speeds. It is also worth noting, that, no known study in the literature has verified either the SWERA or the GWA maps for inland areas of Ghana yet. So, they might not necessarily be more accurate than the WRF map.

## 5 Summary and key conclusions

This study builds on recommendations of previous sensitivity studies of the WRF model for the modelling of wind resources in Ghana. Compared to the previous studies, this study downscaled data to relatively higher temporal and spatial resolutions. The downscaled wind data was verified with ground measurements from seven other locations in addition to the one location that was used in the sensitivity studies.

Results suggest that the WRF model in this configuration generates quite accurate wind speed data. Quite similar conclusions were reached in the sensitivity studies, although they covered relatively short, selected periods [12, 13, 40, 41]. Monthly average wind speeds could not be predicted accurately for all the months, as a "one size fits all" configuration is difficult to obtain in WRF. However, annual average data met most of the performance benchmarks that were used in evaluation. A wind map generated from the averaged downscaled data compares quite better than the existing 1-km wind map for Ghana from 2001 in capturing average wind speeds and its variations in coastal areas in Ghana. The generated spatial map also compares quite well with the unverified higher resolution 250-m map for the coastal regions of Ghana and neighbouring Togo from the Global Wind Atlas.

It is concluded from the results of this study that, the configuration of the WRF model that was used in this study, generates quite accurate data for wind mapping purposes in the coastal areas of Ghana. The configuration also generates quite accurate average annual average wind speeds for the two years this study covered. Given the similarity in local climate and terrain of the coastal areas in neighbouring countries such as Togo and Benin, it is believed that the configuration will probably have similarly good levels of accuracy in those countries, as was seen in the maps for the coastal areas of neighbouring Togo.

Future research should investigate the effect of other driving data sets such as the ERA5 and should cover longer periods of time to better evaluate the model's ability to simulate the wind climate of Ghana. An ensemble system of different configurations that suit different times and conditions to improve the accuracy and consistency of time-series data should also be investigated.

## Declarations

### Author contribution statement

Denis E. K. Dzebre: Conceived and designed the experiments; Performed the experiments; Analyzed and interpreted the data; Wrote the paper.

J. Ampofo: Analyzed and interpreted the data; Wrote the paper.

Muyiwa S. Adaramola: Conceived and designed the experiments; Wrote the paper.

### Funding statement

This work was supported through project titled ‘Upgrading education and research capacity in Renewable energy technologies at Kwame Nkrumah University of Science and Technology (KNUST) Kumasi Ghana’, collaborative project between KNUST and Norwegian University of Life Sciences, Ås Norway, which is funded by Norwegian Agency for Development Cooperation (Norad) through the Energy and Petroleum (EnPe) programme.

### Data availability statement

Data will be made available on request.

### Declaration of interests statement

The authors declare no conflict of interest.

### Additional information

No additional information is available for this paper.
